# Malaysian Patients' Expectations of Orthodontic Treatment in Indian Dental Care Set up: A Questionnaire Survey

**DOI:** 10.1155/2022/1549185

**Published:** 2022-08-23

**Authors:** Ritesh Singla, Nishu Singla, Anuprita Nair

**Affiliations:** ^1^Department of Orthodontics & Dentofacial Orthopaedics, Manipal College of Dental Sciences, Manipal, Manipal Academy of Higher Education, Manipal 576104, Karnataka, India; ^2^Department of Public Health Dentistry, Manipal College of Dental Sciences, Manipal, Manipal Academy of Higher Education, Manipal 576104, Karnataka, India; ^3^Manipal College of Dental Sciences, Manipal, Manipal Academy of Higher Education, Manipal 576104, Karnataka, India

## Abstract

**Materials and Methods:**

A cross-sectional study was conducted on 349 Malaysian patients (182 females and 167 males) aged 18–30 years. A questionnaire consisting of ten items developed by Sawyers and Newton was administered to the patients who visited the department of Orthodontics seeking orthodontic treatment. *Outcome measures.* Descriptive analysis of the responses and comparison of male and female expectations.

**Results:**

Most of the patients expected only a check-up/diagnosis/discussion at their initial appointment, anticipated a fixed type of orthodontic treatment, did not prefer extraction/removal of their teeth, thought the treatment to be painful, and can restrict what they could eat or drink. Positive expectations from the patient included better tooth alignment, enhanced smile, improved confidence, and advancement in professional career. At the same time, nearly half of the patients' assumed speech/mastication does not get affected during the treatment. Moreover, very few patients believed it would be easier to eat/speak/keep their teeth clean after the treatment. Compared to males, more females had significantly anticipated fixed types of orthodontic treatment, thinking the treatment to be painful and restrictive in terms of what they could eat or drink. Most of the females were unsure about the length of the orthodontic treatment and had significantly higher expectations concerning career improvement.

**Conclusions:**

To meet the varied expectations of each of the patients, effective communication between the orthodontist and the patient is essential.

## 1. Introduction

India, with its strong emphasis on the culture of “Atithi Devo Bhava,” has been attracting many overseas students to pursue their education in India for many years. A good number of Malaysian students take up their studies in Indian educational institutions, accounting for 10% of the international student population. Education is an integral part of the multi-faceted relationship between the two countries. Significant twinning arrangements exist between educational institutes in Malaysia and India. Sending students to India for short-term clinical practice training is also a feature at many Malaysian institutes. Many students choose to pursue their higher and professional education in India to get more global exposure during their courses [1].

Similarly, India continues to emerge as one of the world's top dental tourism destinations. Excellent patient care and dental treatment options in India at lower rates than in their respective countries attract many to seek their oral health care needs in India. Hence, many Malaysian students also prefer to seek readily available and affordable dental care during their stay in India for educational purposes. The 2007 National Oral Health Survey of School Children (NOHSS 2007) conducted by the Health Ministry of Malaysia among 16-year-old Malaysian school children reported that 35.3% need orthodontic treatment [2]. Arsalan Sheikh et al. reported Malaysian population aged 18–25 years had Angle's class I malocclusion (48.6%), followed by class III (26.8%), class II (16.2%), and normal occlusion (8.5%) [3]. Hence, there is an increased demand for orthodontic treatment and more awareness amongst these patients. Evaluating patient expectations allows to assess individual patient preferences, needs, concerns, and values and enhancing their satisfaction level.

Their expectations often influence patient satisfaction with the treatment. Expectations are based on anticipated outcomes from the patient perspective, which may or may not be pragmatic. A less preferable result gives rise to the emotion of disappointment. Incomplete expectations from previous treatments may discourage them from seeking care in the future [4, 5]. For patients seeking orthodontic treatment, a lack of understanding of the processes and sequelae can impact patient compliance [6, 7]. Hence, it is important to understand patient expectations and give them a clear picture of the duration, quality of life, and daily changes associated with wearing orthodontic braces. Over the past several years, many studies have correlated orthodontic treatment needs and aesthetics with self-esteem [3, 8, 9]. A study found that the prevalence, extent, and severity of the impact of self-perceived malocclusion in Malaysian adults on oral health-related quality of life were higher than in adults of other nationalities. The most impacted domains were psychological and social impact, esthetic discomfort, and selfconfidence [10].

Hence, clinicians must clearly understand the patient's expectations before initiating treatment. It will help the practitioner to adapt to patient expectations and explain the pros and cons of the treatment to them. Moreover, it would transform their former bad experiences into uplifting ones and encourage them to seek regular oral healthcare in the future. Achieving treatment goals, resolving the chief complaint, and meeting realistic expectations usually lead to favorable final results. Nowadays, the usage of more patient-centered measures assesses the individual characteristics in evaluating orthodontic treatment needs and determining the outcomes of orthodontic care [11–13].

Moreover, a thorough literature search found no such study conducted on this population in the Indian dental health care setup. Therefore, it is the first of its kind to examine Malaysian patients' expectations of orthodontic treatment in an Indian dental care setup. It aimed to know the Malaysian patients' expectations of orthodontic treatment using a reliable and valid questionnaire. A study found that dissatisfaction with the appearance of their dentition and perceived need for braces was more common in females than males [14–16]. Hence, this study also compared the findings between males and females of the study population.

## 2. Materials and Methods

A cross-sectional study comprising 349 young adults (182 females and 167 males) aged 18–30 years, among the Malaysian patients seeking orthodontic treatment at the Department of Orthodontics, Manipal college of dental sciences, Manipal, were selected. Kasturba Medical College and Kasturba Hospital Institutional Ethics Committee, Manipal, provided prior approvals to conduct the student. The inclusion criteria were Malaysian patients aged 18–30 years seeking orthodontic treatment with no previous orthodontic treatment or orthodontic consultation history. In addition, patients having a low intelligent quotient, cleft lip, cleft palate, or any other deformities were excluded.

The participation information sheet explained the purpose and process of the study. The informed consent form highlighted that participation was voluntary. The participant responses were anonymous and confidential. Patients willing to participate and who signed the written informed consent were part of the study. Pre-evaluation revealed a required sample size of 349 students after considering the prevalence at 35% (NOHSS 2007), confidence level at 95% (Z, standard value of 1.96), and margin of error at 5% (d, standard value of 0.05) [2]. Using a purposeful sampling technique, three hundred forty-nine self-administered questionnaires were distributed among the Malaysian patients. The investigators administered the questionnaire, ensured completion, and collected responses from students. The time taken for questionnaire completion was estimated to be 5–7 minutes.

A previously validated questionnaire consisted of ten items developed by Sawyers and Newton to evaluate the patients' expectations [17, 18]. Appropriate permissions from the authors were obtained before starting the study. The questionnaire consisted of questions regarding social demographic information such as age and gender and ten questions measuring the patients' expectation responses on a Likert scale (5-point); the responses ranged from strongly agree to disagree for each statement strongly. Question nos. 8 and 9 had six different categorical response options each. However, based upon the concepts identified in the pilot study done on 30 subjects, subquestions 1(d), 1(e), and 1(f) were removed, and questions 2 and 3 were reworded in the original questionnaire without compromising on its internal consistency. It enabled better understanding by the patients, such as using the term ‘tooth removal' instead of using ‘tooth extracted'. The language used in the questionnaire was English since the subjects were well versed in it.

Data collection occurred from June 2018 to June 2019. The student responses were organized and systematic manner. They were entered and analyzed using SPSS Version 20 (Statistical Package for Social Sciences Corporation, Chicago, USA). The *P* valu*e* < 0.05 was considered statistically significant. Descriptive statistics were employed to describe the responses concerning the patient's expectations. The Pearson's Chi-square test evaluated the differences between males and females.

## 3. Results

Three hundred forty-nine (*n* = 349) patients completed the questionnaire. The mean age of the patients was 24 years old (SD 1.56).  Table 1 depicts the percentage and frequency of responses to the questionnaire items (1–7 and 10). Most patients expected only check-ups and diagnosis (52.4%) or discussion about their treatment (57.3%) at their initial appointment. The majority of the patients (75%) anticipated a fixed orthodontic treatment and did not prefer extraction/removal of their teeth (58.7%). Nearly half of the patients assumed speech (52.7%) or eating (53.3%) would not be affected during the treatment. However, 59.3% of the patients thought it would be painful and could restrict what they could eat or drink (56.1%) during the treatment. Most of the patients expected orthodontic treatment to straighten their teeth (73.7%), produce a better smile (64.1%), improve their chances of having a flourishing career (59%), and get socially confident (60%) after the treatment. On the other hand, few patients believed it would be easier to eat (8.7%) or speak (11.5%) after the treatment. Similarly, nearly half of the patients did not believe it would be easier to keep their teeth clean (48.7%) after the treatment. Moreover, many patients (54.4%) were undecided about the reaction to wearing braces from other people.

Tables 2 and 3 highlight the descriptive statistics for the questions (8 and 9). The majority of the patients (59.1%) did not know how long the orthodontic treatment would last. However, 13.7% believed it to be less than a year, 12.9% thought it to be between 1 and 1.5 years, and very few expected it to be between 2 and 3 years (3.4%) or more than three years (2.3%). Majority of the patients (46.4%) expected to be the frequency of appointments between 1 and 2 months.

On comparison between males and females, it was found that females had significantly lesser expectations of receiving removable orthodontic treatment than males (*p* < 0.0006). More females thought it would be painful (*p* < 0.0001) and could restrict what they ate or drank during the treatment (*p* < 0.0125). Most females did not know how long the treatment would take (*p* < 0.0288) and had significantly higher expectations concerning career improvement (*p* < 0.0001). However, no other statistically significant differences were found in the study.

## 4. Discussion

This study was conducted to find the expectations of orthodontic treatment in Indian dental care set up for Malaysian patients. It was found that most patients had high expectations of orthodontic treatment to improve their aesthetics and social image. Most of the patients in the present study expected that orthodontic treatment would straighten their teeth, produce a better smile, improve their chances of having a promising career, and get socially more confident. Mostly, the perceived benefit of occlusal correction is the ease of maintaining oral hygiene and preventing oral disease [19, 20]. However, in the present study, very few Malaysian patients expected that orthodontic treatment could improve their mastication and speech and can make it easier to keep their teeth clean. These findings indicate that they had meager expectations for improving their oral function. This is per a study that had also reported similar results stating the participants had a minimal expectation of improved oral function [21]. These findings prove that improvement in dentofacial aesthetics is the biggest motivator in a person's decision to undergo orthodontic treatment [22]. Additional benefits seen by patients include improved social life and self-confidence [23].

Orthodontic treatment can sometimes cause pain, discomfort, and functional limitations [5, 23–25]. In the present study, more than half of the patients thought orthodontic treatment would be painful and could restrict what they could eat or drink. Previous studies affirm this as the findings reflect that patients anticipate a considerable amount of discomfort during orthodontic treatment [5, 23, 25, 26]. This may affect patient compliance and satisfaction with treatment and could build up stress between the patient and practitioner [5]. Speech impairment due to wearing braces can affect patients' social confidence. Still, nearly half of the participants in the present study did not expect it to affect speech [27]. Hence, it is crucial that practitioners guide patients on what they should expect from treatment concerning pain, functional limitations, or discomfort.

Male and female patients have different expectations for orthodontic treatment. In the present study, it was found that compared to males, more females anticipated pain during orthodontic treatment. Females had greater expectations of restrictions concerning what they could eat or drink during treatment, had significantly lesser expectations of receiving a removable type of orthodontic treatment, and had significantly higher expectations about career improvement. These findings are similar to those in the Philips et al. study, which reported similar trends in pain, dietary and drinking restrictions, career improvement, treatment time, and type of orthodontic treatment expectations in men and women [28]. Vital social well-being motivated most males, while females focused on improved appearance as their reward. Females report greater dissatisfaction with the appearance of their dentition and a higher perception of orthodontic treatment than males [14–16].

Studies show that wearing orthodontic appliances becomes more acceptable in communities where many children undergo orthodontic treatment [29]. Many patients in the present study were undecided about the reaction to wearing braces from other people. However, a study conducted by Renske Hiemstra et al. found no expectation of negative responses from the public to wearing braces [21]. The majority of the patients in the present study had no perceived idea about the duration of treatment. Orthodontic treatment means time seems to be beyond patients' expectations [30]. Fixed appliance use can negatively impact patients' self-esteem and quality of life; it can be a troubling impedance in their daily routine. Fixed appliances require extra appointments and increased time commitment from the patient. These factors justify that a plausible cause of patient dissatisfaction is the longer than expected treatment time [31]. Providing a more transparent estimate for total treatment time can help provide a more realistic assessment of treatment costs to the patients. Moreover, it will minimize the risk of iatrogenesis and increase success rates and patient satisfaction [30].

Hence, it is necessary to adapt the treatment according to the patient's expectations, replacing their past negative experiences with more positive affirmative ones and promoting oral health. Achieving treatment goals, resolving patients' chief complaints, and meeting realistic patients' expectations generally lead to favorable final results.

The sample of Malaysian patients included in this study was based on a convenient sampling methodology and hence the results are not the representation of the whole Malaysian population in India and should be extrapolated with caution.

## 5. Conclusion

Malaysian patients had high expectations of orthodontic treatment to improve their aesthetics and social image but had relatively low expectations for improving their oral function. Therefore, orthodontists must provide accurate instructions on what patients should expect regarding pain, aesthetics, functional improvement, and discomfort in orthodontic treatment. Bridging the gap between their expectations of health and their experience of it will improve the quality of orthodontic treatment provided to the patient. Employing this strategy in orthodontic patient management will lead to minor disappointment, more satisfied patients, and improved quality of orthodontic care.

## Figures and Tables

**Table 1:** Distribution of the patients according to percentage and frequency of the responses.

Questions		Strongly Agree% (*n*)	Agree% (*n*)	Undecided% (*n*)	Disagree% (*n*)	Strongly disagree% (*n*)
1. At your initial appointment do you expect to:	a) Have a brace fitted?	7.2% (25)	10.3% (36)	15.2% (53)	56.2% (196)	11.2% (39)
	b) Have a check-up and diagnosis?	9.4% (33)	43% (150)	30.4% (106)	10.3% (36)	6.9% (24)
	c) Have a discussion about treatment?	11.5% (40)	45.8% (160)	27.5% (96)	10.3% (36)	4.9% (17)
2. What type of orthodontic treatment do you expect?	a) Removable?	9.1% (32)	12.9% (45)	29.2% (102)	41.5% (145)	7.2% (25)
	b) Fixed?	9.7% (34)	65.3% (228)	10.3% (36)	8% (28)	6.6% (23)
	c) Teeth extracted/Teeth removal?	3.7% (13)	9.2% (32)	28.4% (99)	50.4% (176)	8.3% (29)
	d) Jaw surgery?	0.6% (2)	9.2% (32)	20.3% (71)	40.4% (141)	29.5% (103)
3. Do you expect orthodontic treatment to affect your speech?		10.6% (37)	8.9% (31)	27.8% (97)	39% (136)	13.7% (48)
4. Do you think wearing a brace will be painful?		16% (56)	43.3% (151)	22.1% (77)	9.4% (33)	9.2% (32)
5. Do you think orthodontic treatment will produce problems with eating?		6.3% (22)	10.9% (38)	29.5% (103)	45.3% (158)	8% (28)
6. Do you expect your orthodontic treatment to restrict what you eat or drink?		14% (49)	42.1% (147)	20.6% (72)	12.6% (44)	10.6% (37)
7. Do you think people will react to wearing a brace?		10.6% (37)	13.2% (46)	54.4% (190)	13.5% (47)	8.3% (29)
10. Do you expect orthodontic treatment to:	a) Straighten your teeth?	14.1% (53)	59.6% (197)	17% (61)	7.7% (29)	1.6% (9)
	b) Produce a better smile?	15.7% (59)	48.4% (162)	19.9% (70)	12.2% (55)	3.8% (19)
	c) Make it easier to eat?	2.6% (11)	6.1% (27)	6.4% (30)	63.8% (199)	21.2% (66)
	d) Make it easier to speak?	3.5% (15)	8% (33)	8.7% (36)	59.9% (198)	19.9% (67)
	e) Make it easier to keep your teeth clean?	12.8% (48)	13.8% (52)	24.7% (85)	38.1% (127)	10.6% (37)
	f) Improve your chances of a good career?	7.4% (30)	51.6% (169)	9% (38)	19.2% (68)	12.8% (44)
	g) Give you confidence socially?	22.8% (80)	37.2% (124)	10.9% (42)	13.5% (50)	15.7% (53)

**Table 2:** Patients' expectations as to the duration of orthodontic treatment.

Duration of treatment	Patients% (*n*)
Less than one year	13.7% (48)
1–1.5 years	12.9% (45)
1.6–2 years	8.6% (30)
2–3 years	3.4% (12)
More than three years	2.3% (8)
Don't know	59.1% (206)
Total	100% (349)

(*n* = number of patients).

**Table 3:** Patients' expectations as to the frequency of appointments.

Frequency of appointments	Patients%(*n*)
< 1 month	25.2% (88)
1–2 months	46.4% (162)
> 2–3 months	15.5% (54)
> 3–6 months	2.9% (10)
> 6 months	1.7% (6)
Don't know	8.3% (29)
Total	100% (349)

(*n* = number of patients).

## Data Availability

The study data set is available on request from Ritesh Singla (ritesh.singla@manipal.edu).
